# Göttingen minipig model of diet-induced atherosclerosis: influence of mild streptozotocin-induced diabetes on lesion severity and markers of inflammation evaluated in obese, obese and diabetic, and lean control animals

**DOI:** 10.1186/s12967-015-0670-2

**Published:** 2015-09-22

**Authors:** Trine Pagh Ludvigsen, Rikke Kaae Kirk, Berit Østergaard Christoffersen, Henrik Duelund Pedersen, Torben Martinussen, Jonas Kildegaard, Peter M. H. Heegaard, Jens Lykkesfeldt, Lisbeth Høier Olsen

**Affiliations:** Department of Veterinary Disease Biology, Faculty of Health and Medical Sciences, University of Copenhagen, Ridebanevej 9, 1870 Frederiksberg, Denmark; GLP-1 and Obesity Pharmacology - PK/PD, Novo Nordisk A/S, Novo Nordisk Park, 2760 Måløv, Denmark; Histology & Imaging, Novo Nordisk A/S, Novo Nordisk Park, 2760 Måløv, Denmark; Department of Public Health, Faculty of Health and Medical Sciences, University of Copenhagen, Øster Farimagsgade 5, Postbox 1014 KBH K, Copenhagen, Denmark; Clamp Competency Center, Novo Nordisk A/S, Novo Nordisk Park, 2760 Måløv, Denmark; National Veterinary Institute, Technical University of Denmark, Bülowsvej 27, 1870 Frederiksberg, Denmark

**Keywords:** Atherosclerosis, Animal model, Pig, Cardiovascular disease, Biomarkers, Inflammation, Obesity, Diabetes mellitus

## Abstract

**Background:**

From a pharmacological perspective, readily-available, well-characterized animal models of cardiovascular disease, including relevant in vivo markers of atherosclerosis are important for evaluation of novel drug candidates. Furthermore, considering the impact of diabetes mellitus on atherosclerosis in human patients, inclusion of this disease aspect in the characterization of a such model, is highly relevant. The objective of this study was to evaluate the effect of mild streptozotocin-induced diabetes on ex- and in vivo end-points in a diet-induced atherosclerotic minipig model.

**Methods:**

Castrated male Göttingen minipigs were fed standard chow (CD), atherogenic diet alone (HFD) or with superimposed mild streptozotocin-induced diabetes (HFD-D). Circulating markers of inflammation (C-reactive protein (CRP), oxidized low-density lipoprotein (oxLDL), plasminogen activator inhibitor-1, lipid and glucose metabolism were evaluated together with coronary and aortic atherosclerosis after 22 or 43 diet-weeks. Group differences were evaluated by analysis of variance for parametric data and Kruskal–Wallis test for non-parametric data. For qualitative assessments, Fisher’s exact test was applied. For all analyses, p < 0.05 was considered statistically significant.

**Results:**

Overall, HFD and HFD-D displayed increased CRP, oxLDL and lipid parameters compared to CD at both time points. HFD-D displayed impaired glucose metabolism as compared to HFD and CD. Advanced atherosclerotic lesions were observed in both coronary arteries and aorta of HFD and HFD-D, with more advanced plaque findings in the aorta but without differences in lesion severity or distribution between HFD and HFD-D. Statistically, triglyceride was positively (*p* = 0.0039), and high-density lipoprotein negatively (*p* = 0.0461) associated with aortic plaque area.

**Conclusions:**

In this model, advanced coronary and aortic atherosclerosis was observed, with increased levels of inflammatory markers, clinically relevant to atherosclerosis. No effect of mild streptozotocin-induced diabetes was observed on plaque area, lesion severity or inflammatory markers.

**Electronic supplementary material:**

The online version of this article (doi:10.1186/s12967-015-0670-2) contains supplementary material, which is available to authorized users.

## Background

Cardiovascular complications related to diabetes mellitus are well-known and with a global obesity epidemic, metabolic syndrome including pre-diabetic conditions such as insulin resistance and hyperglycemia has become a major challenge to global health [1]. Despite uncertainty regarding pathophysiological mechanisms, diabetes mellitus is known to be associated with increased cardiovascular risk in humans [2] and proposed to accelerate atherosclerosis in porcine models [3, 4]. Highly comparable cardiovascular anatomy and physiology, combined with a diet-induced plasma cholesterol profile preponderant in pro-atherogenic lipoproteins, render porcine models attractive in research of atherosclerosis [5, 6]. Atherosclerosis develops in similar predilection sites as in humans and several studies indicate development of advanced lesions, highly comparable to human atherosclerosis [3, 7–9]. Recent data from genetically-modified pigs also show advanced lesions [10, 11], however limited availability and substantial cost of these models often restrict their use. Another important consideration in relation to porcine models is size, which often is a challenge in early drug development. The Göttingen minipig is one of the smaller minipig breeds and in addition, studies indicate a greater susceptibility of the breed to develop atherosclerosis as compared to domestic breeds [12]. The breed is well described in relation to glucose metabolism and obesity, with propensity to develop various aspects of the metabolic syndrome including insulin resistance and dyslipidemia [13–15]. Overt diabetes does generally not develop in pigs and induction, e.g. chemically, is therefore necessary [16]. Usually, in studies of diabetic atherosclerotic pigs, long-term sustained hyperglycemia is applied to accelerate the atherosclerosis [4], potentially leading to adverse effects including wasting or deleterious non-thriving of the animals, unless blood glucose is controlled by exogenous insulin [17]. Models presenting a more mild diabetic condition, defined as moderate hyperglycemia, with fasting plasma glucose of approximately 10–15 mmol/L and positive energy balance without exogenous insulin requirement could be relevant in long-term studies [16].

In studies of atherosclerotic pigs, whether diabetes is induced or not, repeated evaluation of atherosclerosis is relevant, especially when investigating potential drug effects. In addition to traditional risk markers, such as elevated total plasma cholesterol and cholesterol fractions, plasma markers of inflammation are becoming increasingly used for improved prediction of cardiovascular disease outcome [18, 19]. Relevant inflammatory markers, e.g. C-reactive protein (CRP) and oxidized low-density lipoprotein (oxLDL), are suspected to be involved in endothelial cell inflammation as well as being mechanistically involved in formation of the atheroma [20, 21]. Other markers, such as tissue plasminogen activator and plasminogen activator inhibitor-1 (PAI-1) relate to hemostasis and impaired fibrinolysis, and especially PAI-1 has been recognized as an indicator in relation to thrombogenicity of atherosclerotic plaques [19, 22]. These may be relevant biomarkers of atherosclerosis in pigs as well as in humans, but need further investigation.

The aim of this study was to evaluate the effect of mild diabetes on aortic and coronary plaque burden and lesion advancement in diet-induced atherosclerotic and mildly diabetic Göttingen minipigs. In addition, the association between plaque burden and circulating metabolic and inflammatory markers, relevant to atherosclerosis was evaluated in animals euthanized after 22 or 43 weeks of atherogenic diet-feeding.

## Methods

### Animals, diet and housing

Castrated male Göttingen minipigs (Ellegaard A/S, Dalmose, DK), were housed at research facilities of University of Copenhagen. Using a staggered design, two cohorts (A and B) with an equal animal distribution in each cohort, mean age 11 weeks at study start, were randomized to three groups; lean control (CD, n = 12) fed a standard minipig diet (Mini-pig, Testdiet, Essex, UK) according to breeders recommendations, and a high-fat/high-cholesterol group (HFD, n = 12) as well as a low-grade diabetic group (HFD-D, n = 12), both fed an atherogenic diet with 2 % cholesterol and 0.7 % sodium cholate (Ossabaw atherosclerotic diet type 5B4L, TestDiet^®^, St. Louis, MO, USA). All animals were fed a total daily amount of 2–2.5 % of bodyweight (evaluated weekly) divided into two daily meals. Animals had free access to water and bedding material and were group-housed, except for periods with intravenous (IV) catheters implanted. Animals from each cohort were euthanized at two different time points: After 22 weeks of diet feeding at a mean age of 33 weeks (CD, n = 6 and HFD, n = 6) or 43 weeks of diet-feeding at a mean age of 55 weeks (CD, n = 6, HFD, n = 6 and HFD-D, n = 12). The study was approved by the Animal Experiments Inspectorate, Ministry of Justice, DK.

Part of the animals in the current study has been described previously in Pedersen et al. [23] and Christoffersen et al. [24].

### Induction of diabetes

Mild diabetes was induced according to previous protocols [17], using a single high IV dose of streptozotocin (125 mg/kg) preceded by IV dosing of nicotinamide (67 mg/kg). One group of animals (all in cohort A) was induced twice, at age 16 and 30 weeks (HFD-D_A_, n = 6), due to waning effect of the first induction, the other group (all in cohort B) was induced once at age 40 weeks (HFD-D_B_, n = 6). For monitoring of glucose (GLU) and fructosamine (FRA) levels, plasma samples were evaluated every 4–6 weeks. Weekly blood GLU was assessed from ear capillary blood, using a hand-held device (Accu-Chek^®^ Aviva Nano, Roche Denmark, Hvidovre, DK) (data not shown).

### In vivo evaluations

Intravenous glucose tolerance tests (IVGTT) were performed before both time points of euthanasia for assessment of intravenous glucose tolerance index (K_G_) and insulin response (area under the curve of insulin, AUC_Insulin_). Body composition was evaluated using dual-energy X-ray absorptiometry (DXA). Plasma samples before euthanasia were evaluated for lipid parameters (triglyceride, TG; total cholesterol, TC; Low-density lipoprotein, LDL; high-density lipoprotein, HDL; very-low density lipoprotein, VLDL) glucose-related measures (FRA and GLU). Inflammatory parameters were evaluated from plasma samples before euthanasia (oxLDL) and serum and plasma samples taken 6 weeks prior to euthanasia (CRP and PAI-1), and analyzed as described below. To avoid potential influence of IV-catheter implantation on CRP and PAI-1, these markers were analyzed from blood sampled prior to IV-catheter implantation.

### Inflammatory parameters

Using a commercially available ELISA-kit (Mercodia AB, Uppsala, Sweden), oxLDL was assessed in plasma, with mouse monoclonal antibodies (mAb 4E6), against a conformational epitope at the oxidized apolipoprotein B100 of the oxLDL [25]. CRP was evaluated from serum, using established protocols, by dendrimer-coupled cytidine diphosphocholine sandwich immunosorbent enzyme-bound assay (ELISA) [26]. PAI-1 was assessed from natriumcitrat-stabilized plasma, using a commercially available ELISA-kit (CSI19905A, Cell Sciences^®^, Canton, MA, USA), with monoclonal anti-human PAI-1 antibody. All samples were run in duplicate, reporting average values [mean coefficient of variation of 5 % (range 0–20 %)].

### Ex vivo assessments: histology and en face evaluations

Following euthanasia, the heart was harvested and the left anterior descending branch of the left coronary artery (LAD) sectioned into transverse segments, for histomorphometric evaluation of plaque burden. Absolute intima area (coronary plaque area, CPA) and relative plaque area (intima/media-ratio, Ratio) were evaluated. En face plaque area (aortic plaque area, APA) was evaluated from the entire aorta. Furthermore, LAD and aorta sections stained with Movat’s pentachrome or Verhoeff Van Gieson’s staining were evaluated qualitatively for lesion severity to differentiate between non-pathological and pathological intimal thickenings, assessed according to modified guidelines from Virmani et al. [10, 27, 28].

Please see Additional file 1: supplementary methods, for further details.

### Statistics

In the statistical models, the purpose was to evaluate overall group differences of all quantitative and qualitative measures and effect of circulating markers on CPA, APA and Ratio.

#### Group differences

*CPA Ratio, APA and circulating markers* Overall group-wise differences of CPA, Ratio, APA and circulating markers were evaluated by use of analysis of variance (ANOVA) with cohort (A, B), period of diet-feeding (22 or 43 weeks) and group (CD, HFD, HFD-D_A_, HFD-D_B_) as class variables. If overall statistical significance was found, specific group differences were analysed using a post hoc Tukey–Kramer test with *p* values adjusted for multiple testing. Residuals were evaluated for normality and transformed accordingly, and with lack of homogeneity of residuals, a Kruskal–Wallis test was applied with Wilcoxon Rank-sum post hoc test.

*Qualitative assessment of LAD and aorta lesions* Based on qualitative findings from histology from LAD and aorta, group differences were evaluated after 43 diet-weeks and illustrated graphically. Furthermore, to assess effect of study duration on aortic and LAD lesion progression, non-atherosclerotic intimal lesions and progressed atherosclerotic lesion findings were evaluated in HFD only, after 22 and 43 weeks of diet-feeding. Group-wise differences were evaluated using Fishers exact test with p < 0.05 considered significant.

#### Circulating markers association with CPA, APA and Ratio

*Individual markers* To evaluate the effect of circulating markers on CPA, Ratio and APA, each marker was individually included in an ANOVA with cohort, study duration and group as class variables, and the model backwards reduced stepwise, until only significant findings remained.

*Biologically-related markers* To avoid excess explanatory variables in one statistical model, the effect of biologically-related markers on CPA, Ratio and APA were evaluated in groups using multiple linear regression analyses: inflammatory (CRP, oxLDL and PAI-1), glucose metabolism (GLU, AUC_Insulin_, FRA and K_G_) and lipid markers (TG, VLDL, HDL and LDL). All parameters from each group were included in a model, including the abovementioned class variables, with stepwise backwards reduction until only significant findings were left. Data were transformed when appropriate, in order to achieve homogeneity of residuals.

All statistical analyses were performed in SAS 9.2 (SAS Institute Inc., Cary, NC, USA) and graphical illustrations in GraphPad Prism (version 6.04, GraphPad Software Inc., La Jolla, CA, USA). A *p* value <0.05 was considered statistically significant.

## Results

Animal background characteristics are presented in Table 1. One animal died prematurely from unknown cause (HFD-D_B_, n = 1), another showed severe vascular inflammation, diagnosed histopathologically as polyarteritis nodosa, inconsistent with studies of atherosclerosis (HFD, n = 1). In HFD and HFD-D enlarged pale livers were observed at euthanasia when comparing to CD. In previous studies, applying a similar diet to pigs, hepatic macro- and microscopical changes have been observed, comparable to non-alcoholic steatohepatitis in humans [29].

**Table 1:** Background characteristics of animals in the study

Group	CD		HFD		HFD-D_A_	HFD-D_B_
Study duration	22 weeks (n = 6)	43 weeks (n = 6)	22 weeks (n = 6)	43 weeks (n = 5)	43 weeks (n = 6)	43 weeks (n = 5)
Body weight (kg)	15.8 (12.8–17.5)	24.0 (23.1–24.5)	23.8 (22.8–25.4)	53.5 (50.7–60.1)	52.5 (48.4–53.4)	56.6 (54.3–58.3)
Body fat/body weight (%)	10.4 (8.5–12.8)	17.9 (15.6–19.7)	28.3 (27.8–29.6)	49.3 (47.3–51.5)	50.3 (48.6–50.8)	51.3 (49.0-51.7)

Median and interquartile range (25th–75th)

*CD* control animals, *HFD* high-fat/high-cholesterol fed animals, *HFD-D*_*A*_ *and*_*B*_ high-fat/high-cholesterol fed diabetic animals group A and B

### Mild diabetes

**Fig. 1:**
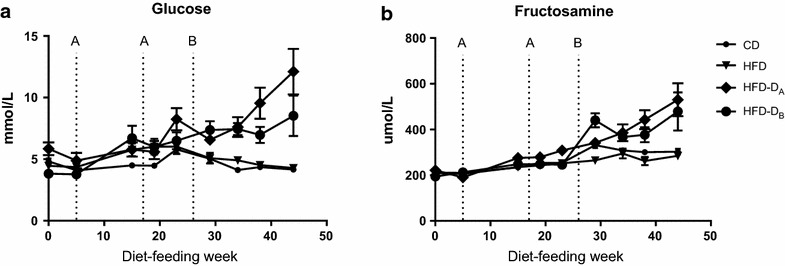
Illustration of plasma glucose (**a**) and fructosamine (**b**) over time in non-diabetic animals [lean control (CD) and high-fat/high-cholesterol fed (HFD)] compared to the two diabetic groups (HFD-D_A_, HFD-D_B_). Diabetes was induced twice in HFD-D_A_; after 5 and 17 diet-feeding weeks and once in HFD-D_B_, after 26 diet-feeding weeks. Diabetes induction is illustrated by ticks at each time point, marked by *A* and *B*, respectively. Data is shown as mean ± SEM. Please note, not all animals are represented at each time point

In Fig. 1, the plasma GLU and FRA levels over time are graphically illustrated, with HFD-D_A_ and _B_ compared to CD and HFD, illustrating increased fasting levels of both parameters in the two diabetic groups over the course of the study. The pigs did not receive any exogenous insulin and furthermore, no BW difference between obese groups was observed, as seen in Table 1.

### Group differences

#### Circulating markers, CPA, Ratio and APA

**Fig. 2:**
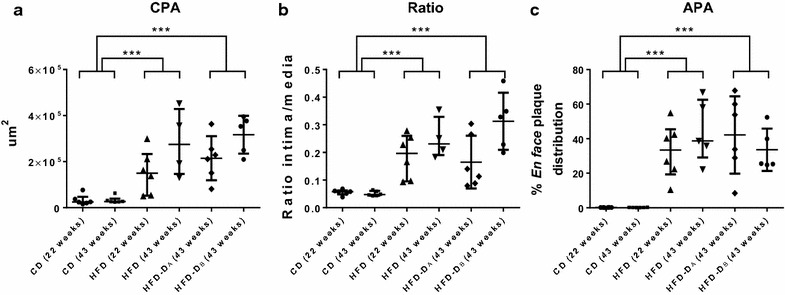
Plaque burden in lean control (CD), high-fat/high-cholesterol fed [HFD (one outlier removed for CPA and Ratio at 43 diet-weeks)] and high-fat/high-cholesterol fed and diabetic animals (HFD-D_A_, HFD-D_B_). **a** Coronary plaque area (CPA), in left anterior descending artery (LAD), **b** intima/media-ratio (Ratio) in LAD, **c** aortic plaque area evaluated *en face* (APA). Median and interquartile range (25th–75th). *P* value <0.05 was considered significant. ***p value <0.001

**Fig. 3:**
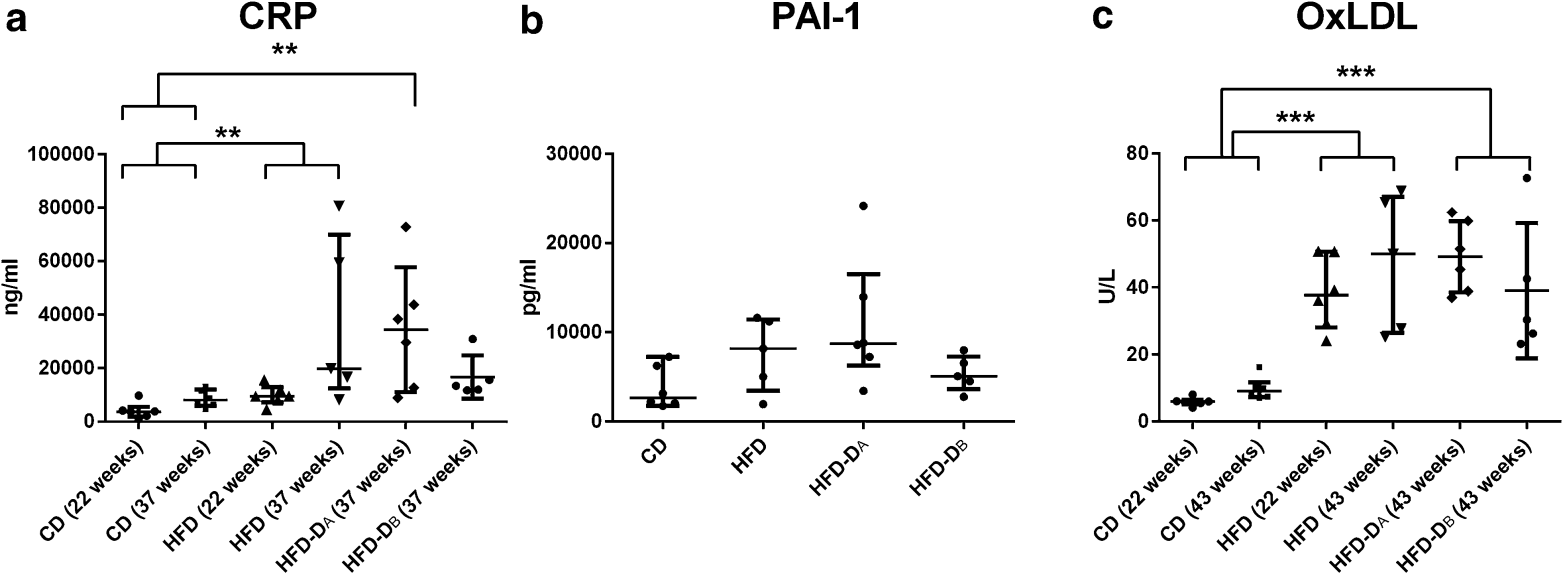
Inflammatory markers in lean control (CD, n = 12), high-fat/high-cholesterol fed (HFD, n = 11) and high-fat/high-cholesterol fed and diabetic animals (HFD-D_A_, n = 6; HFD-D_B_, n = 5). **a** C-reactive protein (CRP), **b** plasminogen activator inhibitor-1 (PAI-1) (only evaluated after 37 diet-weeks), **c** oxidized low-density lipoprotein (oxLDL). Median and interquartile range (25th–75th). Please note, part of data from **a** has been presented in other context [24]. P value <0.05 was considered significant.**p value <0.01, ***p value <0.001

No significant effect was observed of the class variable cohort on any of the response variables, however a significant effect of the class variable study duration (22 or 43 weeks) was observed for insulin (AUC_Insulin_) response to the IVGTT (Table 2). Results from group differences of CPA, Ratio and APA are illustrated graphically in Fig. 2, with significantly increased plaque burden in the left anterior descending artery (LAD) and *en face* in the aorta, in HFD and HFD-D compared to CD. One animal (HFD) displayed severe coronary occlusion compared to the remaining and was defined as a statistical outlier in the residual plots and therefore excluded from analyses with CPA and Ratio as response variables. Figure 3 illustrates group differences of inflammatory markers, with oxLDL significantly increased in HFD and HFD-D, compared to CD. Overall significant group difference was found for CRP, however, only CRP of HFD and HFD-D_A_ was significantly increased compared to CD. No significant differences in PAI-1 among groups were found, but a tendency towards increased levels in HFD and HFD-D compared to CD was observed. Group differences of glucose metabolism are shown in Table 2. GLU and FRA were significantly increased in HFD-D as compared to HFD and CD. A significantly lower AUC_Insulin_ was observed for HFD-D as compared to lean CD and HFD. In HFD after 43 diet-weeks, a significantly increased AUC_Insulin_ was observed compared to CD (*p* = 0.025), suggesting some degree of insulin resistance in HFD. Glucose clearance, assessed by the intravenous glucose tolerance index (K_G_) was decreased in both diabetic sub-groups compared to CD and HFD in diet-week 43, and for HFD-D_A_ compared to CD in diet-week 22. Despite one sub-group (HFD-D_A_) thus displaying slightly more impaired glucose metabolism, an overall significantly altered glucose metabolism was observed for the HFD-D as compared to CD and HFD. In relation to plasma lipids, HFD and HFD-D were as expected significantly more dyslipidemic compared to CD, with moderate triglyceridemia and severe hypercholesterolemia (Table 2) and with no significant difference in hypercholesterolemia between the two HFD-groups. In HFD and HFD-D, the main part of the cholesterol consisted of VLDL although LDL also was elevated compared to CD (Table 2). HDL was increased, corresponding to previous porcine studies [3, 10].

**Table 2:** Group differences of circulating lipid and glucose markers

Group	CD		HFD		HFD-D_A_	HFD-D_B_	Overall p value
Study duration	22 weeks (n = 6)	43 weeks (n = 6)	22 weeks (n = 6)	43 weeks (n = 5)	43 weeks (n = 6)	43 weeks (n = 5)	
GLU^a^ (mmol/L)	5.6^A^ (5.3–5.9)	3.9^A^ (3.6–4.1)	6.2^A^ (4.9–6.4)	4.0^A^ (3.9–4.1)	14.4^B^ (9.4–15.2)	8.0^C^ (6.1–8.3)	<0.0001
FRA^b^ (µmol/L)	257^A^ (251–269)	226^A^ (210–237)	251^A^ (229–266)	242^A^ (234–252)	410^B^ (298–444)	340^C^ (322–387)	0.0004
K_G_^a,c^ (min^−1)^	4.5^AB^ (3.2–5.1)	5.2^A^ (4.9–6.3)	2.8^AB^ (2.4–3.2)	3.4^AB^ (2.4–5.3)	1.2^C^ (0.4–1.8)	1.4^BC^ (1.1–1.5)	<0.0001
AUC_insulin_^c^ (pM min)	4299^A^ (3173–7746)	6943^A^ (6356–7399)	5194^A^ (3676–6236)	11,506^B^ (10,503–12,714)	1992^A^ (1669–4937)	4244^A^ (3368–6747)	0.0003
TG (mmol/L)	0.43^A^ (0.35–0.50)	0.34^A^ (0.29–0.54)	1.09^B^ (1.03–1.36)	1.26^B^ (0.75–2.13)	1.42^B^ (1.26–2.65)	0.92^C^ (0.9–1.45)	0.008
TC^a^ (mmol/L)	1.77^A^ (1.46–2.05)	1.85^A^ (1.53–2.44)	21.70^B^ (19.93–26.76)	16.95^B^ (11.64–25.87)	19.53^B^ (16.59–22.56)	18.87^B^ (17.64–19.18)	<0.0001
VLDL^b^ (mmol/L)	0.07^A^ (0.04–0.10)	0.14^A^ (0.05–0.69)	13.66^B^ (9.49–14.62)	12.50^B^ (7.60–22.32)	12.30^B^ (10.35–14.19)	10.25^B^ (9.58–11.58)	<0.0001
LDL^a^ (mmol/L)	0.72^A^ (0.51–0.85)	0.67^A^ (0.64–1.31)	6.72^B^ (5.12–10.68)	6.22^B^ (4.05–6.57)	5.42^B^ (4.16–6.33)	6.21^B^ (6.01–6.43)	<0.0001
HDL^a^ (mmol/L)	1.00^A^ (0.89–1.11)	0.82^A^ (0.23–1.06)	2.96^B^ (2.60–3.41)	1.26^B^ (1.17–1.38)	1.16^B^ (0.97–1.56)	2.05^B^ (1.45–2.19)	<0.0001

Median and interquartile range (25th–75th)

Values sharing the same superscript letter are not statistically different. A *p* value <0.05 is considered significant

*CD* control animals, *HFD* high-fat/high-cholesterol fed animals, *HFD-D*_*A*_ *and*_*B*_ high-fat/high-cholesterol fed diabetic animals group A and B, respectively, *GLU* glucose, *FRA* fructosamine, *K*_*G*_ intravenous glucose tolerance index, *AUC*_*Insulin*_ area under the curve of insulin, *TG* triglycerides, *TC* total cholesterol, *VLDL* very-low density lipoprotein, *LDL* low-density lipoprotein, *HDL* high-density lipoprotein

^a^Log-transformed data

^b^Non-parametric data evaluated in Kruskal–Wallis test

^c^N = 3 (HFD-D) were excluded due to catheter failure for the intravenous glucose tolerance test (IVGTT)

#### Qualitative assessment of LAD and aorta

**Fig. 4:**
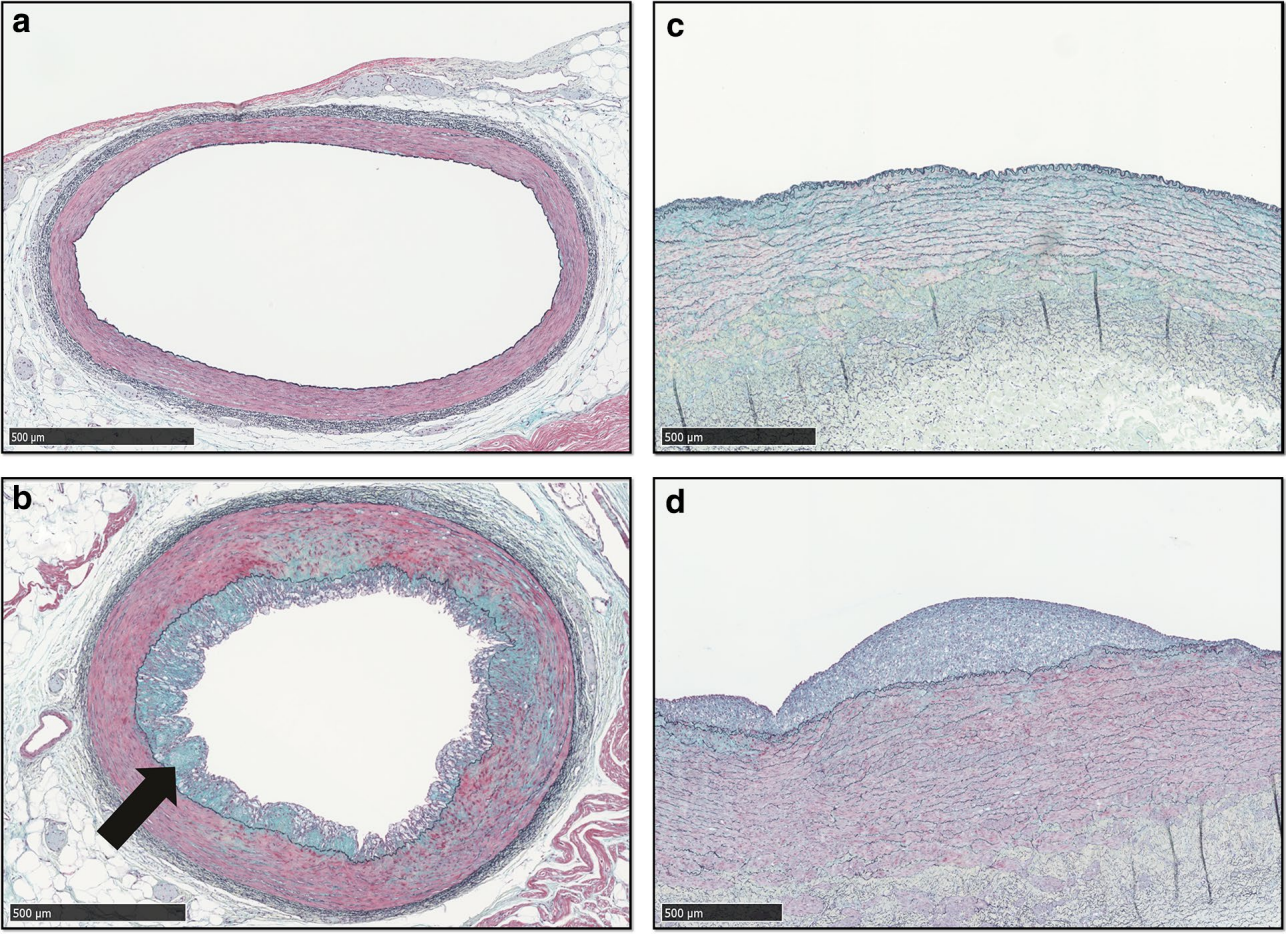
Examples of non-pathological intimal lesions in left anterior descending artery (LAD) and abdominal aorta. Movat’s pentachrome staining. *Scale bar* 500 µm. **a** LAD, lean control animal (CD). **b** LAD, high-fat/high-cholesterol fed animal (HFD); xanthoma with proteoglycan-rich matrix (*arrow*). **c** Aorta, CD. **d** Aorta, HFD; xanthoma

**Fig. 5:**
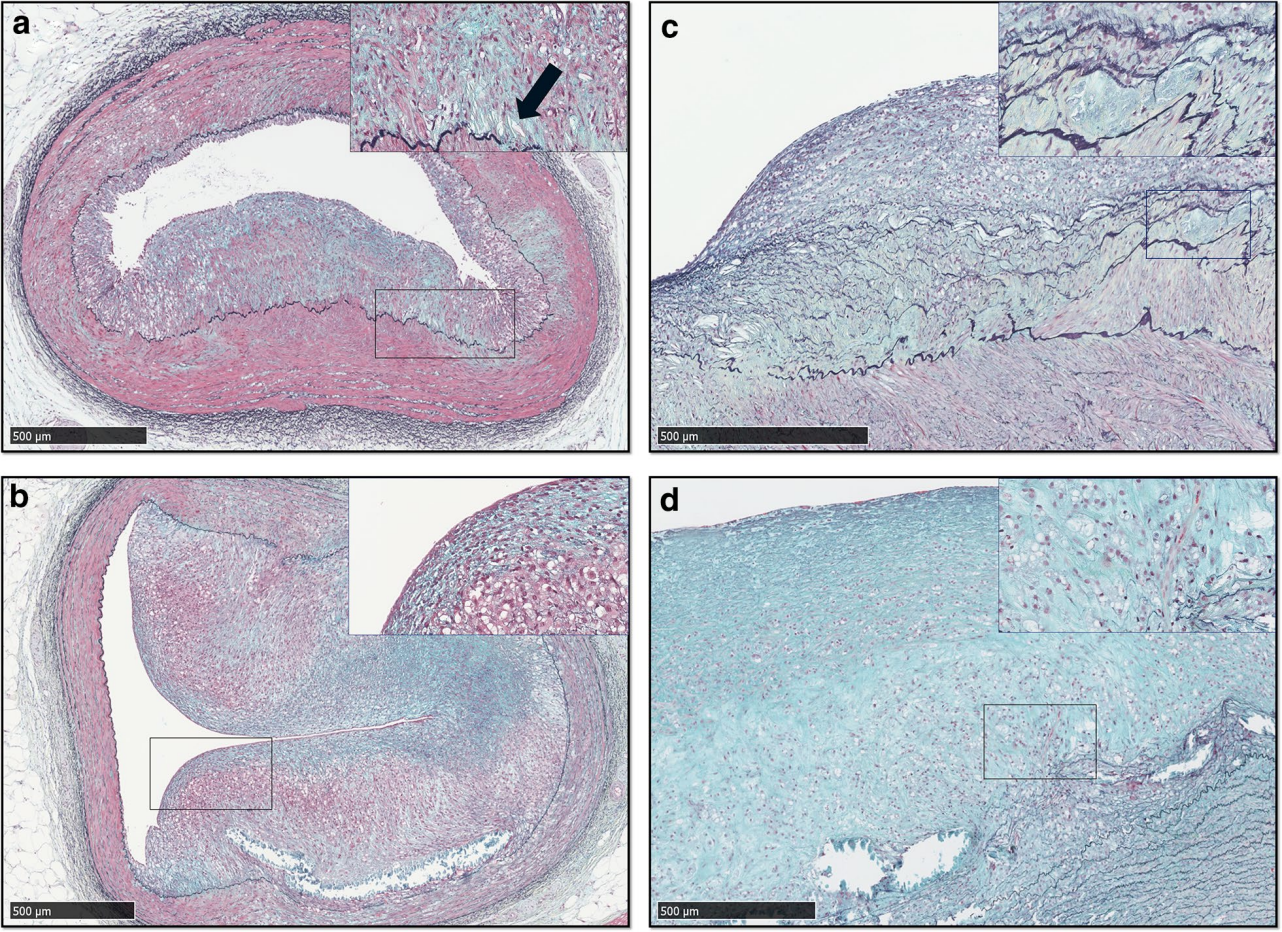
Examples of progressed atherosclerotic lesions in left anterior descending artery (LAD) and abdominal aorta. Movat’s pentachrome staining. *Scale bar* 500 µm. **a** LAD, high-fat/high-cholesterol fed animal (HFD), pathological intimal thickening. *Inset* cholesterol clefts and calcification. **b** LAD, HFD, fibroatheroma with marked calcification. *Inset* cap of the lesion. **c** Pathological intimal thickening in aorta. *Inset* area of calcification. **d** Fibroatheroma in aorta. *Inset* cholesterol clefts disrupted internal elastic lamina and angiogenesis

**Fig. 6:**
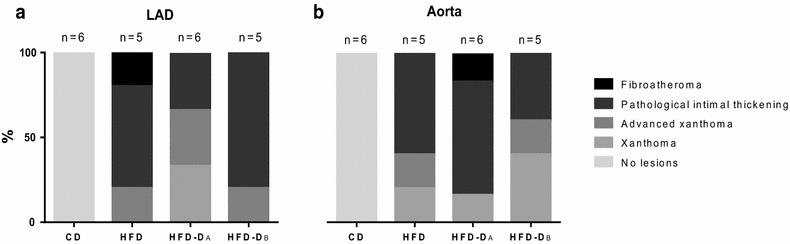
Distribution of intimal lesions in left anterior descending artery (LAD) (**a**) and aorta (**b**) from control (CD), high-fat/high-cholesterol fed (HFD) and high-fat/high-cholesterol fed diabetic animals, group A or B (HFD-D_A_ or _B_), diet-week 43

Progressive atherosclerotic lesions were observed in both aorta and LAD from HFD and HFD-D, whereas no lesions or non-pathological intimal thickening was observed in CD (Figs. 4, 5). Histologically, in LAD, lesions in HFD and HFD-D spanned from xanthoma to fibroatheroma (Figs. 4, 5, 6). Both simple as well as advanced xanthomas, the latter characterized by presence of calcification and/or fibrous tissue, were observed in LAD from HFD and HFD-D. Fibroatheroma, with severe enlargement of the intima, presence of calcification, extracellular lipids and marked fibrous tissue deposition, was observed in LAD of one HFD animal (Fig. 6). Although areas of necrosis were observed, overt necrotic core, defined as total absence of matrix [30], was generally not observed in LAD and pathological intimal thickenings dominated the findings [27]. Cholesterol clefts were occasionally observed in LAD, but more frequently in the aorta. In the aorta, beside the findings described above, angiogenesis was also observed. In the LAD, a trend towards HFD displaying more advanced plaque than HFD-D (*p* = 0.063) was observed, whereas the opposite was observed in the aorta (*p* = 0.063). No effect of time was observed for lesion advancement in the aorta (*p* = 0.82), whereas a trend was observed for the LAD (*p* = 0.067) (Table 3).

**Table 3:** Study duration effect on LAD lesion severity in high-fat/high-cholesterol fed animals (HFD) at diet-week 22 (n = 6) and 43 (n = 5)

Diet week	Non-atherosclerotic intimal lesions	Progressive atherosclerotic lesions	N total
22	5	1	6
43	1	4	5
Total	6	5	

Graduation of lesions according to Virmani et al. [27]. No statistical significant difference was observed in Fisher’s exact test (significance level p < 0.05)

*LAD* left anterior descending artery, *HFD* high-fat/high-cholesterol diet fed animals

### Circulating markers association with CPA, Ratio and APA

Individually evaluated effect of circulating markers (CRP, oxLDL, PAI-1, GLU, FRA, AUC_Insulin_, K_G,_ TG, VLDL, HDL and LDL) on CPA, Ratio and APA, only revealed a significant association between APA and oxLDL (*p* = 0.015). In relation to biologically-related grouped markers effect on plaque burden, a positive association was observed between CPA and oxLDL (*p* = 0.024) and PAI-1 (*p* = 0.048). However, no corresponding association was observed between inflammatory markers and Ratio. No effect of grouped glucose parameters was observed. For grouped lipid parameters, TG was positively (*p* = 0.0039) whereas HDL was negatively associated with APA (*p* = 0.0461). No effect of lipid parameters was found on CPA or Ratio. Group effect remained significant in all the reduced models.

Please see Additional file 2: supplementary results, for further details.

## Discussion

From a pharmacological point of view, a globally-available small-sized pig breed, well-characterized in relation to genetics, microbiology, toxicology as well as lipid and glucose metabolism has potential advantages over existing pig models in relation to drug development. In this study, such a minipig model was evaluated, displaying progressed atherosclerotic lesions in both coronary arteries and aorta after high-fat/high-cholesterol feeding. In the model, no effect of mild chemically-induced diabetes was observed in relation to pathology, circulating inflammatory or lipid markers. The diabetic animals displayed impaired glucose metabolism, without wasting or need for exogenous insulin treatment, suggesting a mild condition of diabetes with no body weight differences observed between diabetic and non-diabetic obese animals. The observed divergence between the two diabetic groups in terms of glucose tolerance and metabolism, was probably due to the difference in diabetes induction, with repeated induction in one group (HFD-D_A_) and single induction in the other group (HFD-D_B_). Heterogeneous outcome from chemically-induced diabetes is a well-known challenge, despite standardization of protocols and animals [17]. In our study, no effect of mild diabetes was observed on dyslipidemia. In human patients, diabetic dyslipidemia with low HDL, preponderance in pro-atherogenic dense LDL particles, and marked triglyceridemia is explained partly by glucose-mediated imbalance in hepatic lipid metabolism. However, presence of excess plasma insulin, as observed in insulin resistant patients, also contributes to this effect [30]. Although the diabetic minipigs displayed impaired glucose metabolism, the absence of hyperinsulinemia is a shortcoming in this diabetic model. The overall lack of difference between diabetic and non-diabetic animals in the current study was interesting, considering previous studies, reporting significant differences between diabetic and non-diabetic animals both in relation to pathology and some circulating plasma markers, in particular TG [3, 31]. However, the animals were considerably more hyperglycemic in these previous studies compared to the present study, which could explain part of the dissimilarity. Interestingly, the pathology findings reported here are in agreement with a recently published paper of a genetically-modified pig model of atherosclerosis displaying no progression in atherosclerosis with poor glycaemic control [32]. In that study, plasma glucose levels were higher compared to the present study, with requirement for exogenous insulin. Diabetic and non-diabetic animals displayed only a slight weight difference and no differences in cholesterol levels. This could support that a relatively well-controlled experimental diabetic condition does not result in more advanced atherosclerotic lesions, at least not within a 1 year study period, as observed in our and the study of Al-Mashhadi et al. [32].

In relation to vascular pathology, the progressed atherosclerotic lesions observed were predominantly pathological intimal thickenings in both HFD and HFD-D. Advanced traits, such as necrosis and angiogenesis, were observed more frequently in the aorta compared to the LAD, apparently independent of the induced diabetes. This finding is known in human patients [33], however of less clinical importance, considering the consequences of an atherothrombotic event in the aorta compared to the coronary or cerebral arteries. In a recent publication, a similar finding was reported from a domestic pig model of atherosclerosis, with more advanced findings in the aorta compared to the coronary artery [34]. Despite this observation, an increased inflammatory gene expression was observed in the coronary arteries, compared to the aorta in this study. Another finding associated with progressed atherosclerosis, frequently observed in the LAD in the Göttingen minipig model, was deposits of calcium at the basal part of the intima, adjacent to the internal elastic lamina. While this finding is comparable to other porcine studies [3, 10, 28], it is phenotypically divergent from typical human lesions [35]. In humans, calcification is often observed adjacent to a necrotic lipid core as a component of the overlying fibrous cap [27, 30]. In the minipig model, calcification was in some sections associated with otherwise minimally affected areas. These observations were therefore considered advanced xanthoma, rather than pathological intimal thickening. Pathogenesis of calcification in atherosclerosis remains unclarified [27, 35], and the phenotype observed experimentally could reflect the accelerated lesion development in these models compared to human patients. Evaluation of plaque burden by CPA and Ratio, corresponded to previous findings [11], with no statistically significant effect of mild diabetes or study duration on lesion area. Evaluation of difference over time in lesion severity was challenged by small sample sizes, and only a tendency for the lesions to be more advanced with time was observed in the LAD.

Considering previous findings of a propensity of castrated animals to develop more metabolic disturbances [14], animals in the current study were castrated. An atheroprotective effect of sex hormones is recognized in humans [36], and studies indicate that female minipigs are more prone to develop coronary atherosclerosis, having a sex hormone profile corresponding to that of castrated males [14, 37]. The effect of castration on atherosclerosis was however not systematically evaluated in our study. The dyslipidemia observed in both HFD and HFD-D, corresponded to previous reports in atherogenic diet-fed pigs, with VLDL as the predominant cholesterol fraction [10, 36]. The important role of TG-enriched larger-sized lipoprotein particles (VLDL, intermediate-density lipoprotein, chylomicron) as biomarkers and in terms of atherogenicity, is becoming increasingly recognized [38]. Scavenger receptors in intimal foam cells might bind these particles without oxidative modification, in contrast to LDL [39]. Combined with the ability to exert vascular inflammation and endothelial dysfunction, the atherogenic potential of these particles is becoming evident [38, 39]. In our study, no direct association was observed between TC, LDL, HDL, VLDL and CPA or Ratio, whereas a negative association was observed between HDL and APA. Atherosclerosis in pig models is also observed at more moderate plasma cholesterol levels [40, 41], and the lack of direct correlation of plaque area and plasma levels of cholesterol is a previously reported finding [28, 42], most likely reflecting a complex relation between plaque area and plasma cholesterol. An association between TG and APA was observed in our study and although plaque lipid composition was not evaluated, the Sudan IV staining applied to the aorta dissolves in TG potentially contributing to the correlation observed.

In relation to inflammatory markers, availability of relevant antibodies is still a challenge, despite increased use of pigs in biomedical research. In this study, three relevant markers were evaluated. The overall increased levels of oxLDL correspond to previous findings in pigs, with circulating oxLDL evaluated in relation to early atheroma formation and in diet-withdrawal studies [40, 43]. Although a positive association was found between APA, CPA and oxLDL, this was not observed between Ratio and oxLDL and precaution should therefore be taken when interpreting the influence of oxLDL on aortic or coronary plaque burden.

A more than threefold increase in levels of CRP was observed in HFD and HFD-D, compared to CD whereas CRP levels observed in human patients in relation to cardiovascular risk, are two to threefold increased (>3 mg/L) [44]. Levels of CRP assessed in patients are subtle fluctuations in the high end of the normal range, and considered indicative of low-grade inflammation, whereas CRP levels >10 mg/L in humans are associated with acute inflammation [44]. In our study, some pigs displayed high levels of CRP, corresponding to previous findings in experimentally-induced acutely inflamed animals [45], suggesting a higher grade of inflammation as compared to humans. Most likely this observation could reflect the accelerated disease state in this model. In humans, increased body weight has been associated with increased CRP levels [46], but in our study, the significant effect of group and study duration was still present when correcting for body weight (data not shown). Previous porcine obesity models fail to display increased inflammation when comparing to lean control animals [47–49]. The dietary content of fructose in the present study could be an important contributor to the inflammatory state observed in the present and other studies applying a similar fructose-enriched high fat-diet [29]. In humans, added dietary fructose has been associated with increased fatty liver disease [50], suggested to play an important role in development of metabolic syndrome and systemic inflammation [50]. Whether the inflammatory state observed in the current and previous studies is a consequence of a pathological liver condition, remains to be further investigated. The variation in inflammatory state in different porcine obesity models is an important consideration from a translational point of view, obviously encouraging thorough considerations on the dietary composition and approach in obesity models [51]. Increased CRP levels have beside obesity also been associated with other aspects of metabolic syndrome in humans such as hyperglycemia and insulin resistance [19, 46] Although no effect of diabetes was observed on CRP levels in the present model, these are relevant considerations when evaluating the predictive value of CRP, specifically in relation to atherosclerosis in this model.

Although a tendency was observed for PAI-1 to differ between groups, no significant differences were observed, in agreement with previous findings in pigs [52].

Besides the aforementioned variability in groups in terms of diabetes induction, an important limitation in this study is the number of animals included, in particular in relation to evaluation of the association between biomarkers and pathology. This, however, is often a limitation when working with advanced porcine models. Cholate was added to the diet to inhibit endogenous conversion of cholesterol to bile acids, however murine studies suggest a pro-inflammatory effect of cholate [53, 54]. Data on systematic evaluation of this effect in pigs are sparse and a potential pro-inflammatory effect cannot be excluded [55]. Scoring of plaque advancement was based on morphology alone, whereas e.g. immunohistochemical characterization of cell populations or inflammation could be highly relevant. Furthermore, lesion severity in relation to circulating markers was not assessed, due to small sample sizes. Both are relevant aspects for further evaluation in the model. Luminal diameter was not a considered end-point in this study. It could be relevant in future studies, in addition to the already presented end-points, but requires perfusion fixation under physiological conditions, which was not executed in the current study. Blood pressure is a risk factor for atherosclerosis, but not assessed in the present study, however in pigs fed a similar diet, mild hypertension was observed [56]. Considering previous findings in pigs fed a similar diet, liver histology could be relevant to evaluate in the model [29].

## Conclusion

In conclusion, a diet-induced minipig model of advanced atherosclerosis has been characterized, including several translational plasma markers for evaluation of inflammation. Induction of mild diabetes did not accelerate atherosclerosis, however, in order to evaluate an add-on diabetes effect of a cardiovascular drug in the model, diabetes induction could still be relevant.

## Additional files

**Additional file 1.** Supplementary methods.
**Additional file 2.** Supplementary results.

## Abbreviations

APA: aortic plaque area
AUC_Insulin_: area under the curve of insulin
CPA: coronary plaque area
CRP: C-reactive protein
DXA: dual-energy X-ray absorptiometry
FRA: fructosamine
GLU: glucose
HDL: high-density lipoprotein
HFD: high-fat/high-cholesterol fed animals
HFD-D: high-fat/high-cholesterol fed and diabetic animals
IVGTT: intravenous glucose tolerance test
K_G_: intravenous glucose tolerance index
LDL: low-density lipoprotein
OxLDL: oxidized LDL
PAI-1: plasminogen activator inhibitor-1
Ratio: intima/media-ratio
TC: total cholesterol
TG: triglyceride
VLDL: very-low density lipoprotein
