# Supplementary material for: Göttingen minipig model of diet-induced atherosclerosis: influence of mild streptozotocin-induced diabetes on lesion severity and markers of inflammation evaluated in obese, obese and diabetic, and lean control animals
Source: J Transl Med. 2015 Sep 22;13:312. doi: 10.1186/s12967-015-0670-2 (PMC4580291; doi:10.1186/s12967-015-0670-2)
Supplement: Supplementary file 1 — Additional file 1. Supplementary methods. [file 12967_2015_670_MOESM1_ESM.docx]

**Additional file 1: Supplementary methods**

*Anesthesia*

For all procedures requiring anesthesia, the animals were anesthetized according to the same protocol; an IM dose of a tiletamin and zolazepam mixture (0.81 mg/kg of both tiletamin and zolazepam) (Zoletil 50 Vet, ChemVet, Silkeborg, DK), with added ketamine (0.81 mg/kg) (Ketaminol Vet (100 mg/ml) Intervet, Skovlunde,DK), xylazine (0.84 mg/kg) (Rompun Vet (20 mg/ml) Bayer, Lyngby, DK) and buthorphanol (0.16 mg/kg) (Torbugesic (10 mg/ml) Scanvet, Fredensborg, DK) was applied for procedures requiring anesthesia.

*Implantation of intravenous (IV) catheters*

Implantation of IV catheters was done approximately one week prior to the procedures necessitating IV access (induction of diabetes and intravenous glucose tolerance test (IVGTT)). Using Seldingers technique, the auricular vein was punctured and the end of the catheter positioned in the jugular vein (BD Careflow, 3 Fr, Argon Medical Devices Inc., Holte, DK). Alternatively, using ultrasound equipment (Vivid *I*, GE Healthcare, Brøndby, DK), the jugular vein was visualized for guidance of positioning of the catheter in this vein (Certofix Protect Duo V730, 7 Fr, B. Braun Medicals, Frederiksberg, DK).

*Body composition scan*

Body composition was evaluated in anesthetized animals using dual-energy x-ray absorptiometry (DXA) (Lunar Prodigy, GE Healthcare, Brøndby, DK).

*Blood sampling*

Blood was sampled before euthanasia from awake and over-night fasted animals using either the cranial caval vein by the BD Vacutainer^TM^ blood collection system (BD medicals Europe, Albertslund, DK) or if functional, the IV catheters. An exception to this was plasma for plasminogen activator inhibitor-1 (PAI-1) and the last time point for measure of C-reactive protein (CRP). Both were sampled six weeks prior to the euthanasia (diet-week 37). After sampling, ethylenediaminetetraacetic acid (EDTA) and natriumcitrate-stabilized plasma was centrifuged within 15 minutes at 4ºC for 10 minutes at 2000 g. In addition, blood in uncoated tubes was left to clot for 1 hour at room temperature before centrifugation. Plasma for clinical biochemistry was stored at -20ºC and remaining samples at -80ºC. Fructosamine (FRA), glucose (GLU) and triglyceride (TG) were measured from EDTA-stabilized plasma using standard equipment (Cobas®6000, Roche Diagnostics A/S, Hvidovre, DK).

*IVGTT*

Baseline blood samples were collected 15 minutes (-15 min) and immediately before (0 min) infusion of glucose (0.3 g/kg) (500 g/L glucose, SAD, DK), in awake and unrestrained animals. This was followed by collection of blood at 1, 3, 5, 7, 10, 15, 20, 25, 30, 35, 40, 50, and 60 minutes post-dosing. All samples were distributed into tubes containing protease inhibitor (250 KIU/ml of aprotinin, Trasylol®, Bayer HealthCare Lyngby, DK) and EDTA (8 mmol/L). Samples were centrifuged at 2000 g at room temperature, within 1 hour after sampling and 10 µL of plasma analyzed for glucose, using the immobilized glucose oxidation method with plasma distributed into 500 µL buffer (EBIO plus, autoanalyzer and solution; Eppendorf, Germany). Remaining samples were stored at -20ºC until analyzed for insulin, using previously described protocols.[1] IV glucose tolerance index (K_G_) was calculated as the negative slope of the linear regression of the natural logarithm to glucose within the 5-30 min interval and area under the curve for insulin response was calculated at sampling time 0-60 minutes (AUC_Insulin­_) [2].

*Total cholesterol (TC) and cholesterol fractions*

TC was evaluated from EDTA-stabilized plasma, by colorimetry (SpectraMAX 250, Molecular Devices, Sunnyvale, California, US) using cholesterol standard (C.f.a.s, Roche Diagnostics, Indianapolis, Indiana, US). Fractionation of lipoproteins was performed by high performance liquid chromatography (HPLC), using Superose 6 columns (10/300, GE Healthcare, Brøndby, DK) in an automatized system (Agilent 1100 HPLC system, Agilent Technologies, Waldbronn, GE). Plasma samples of 100 µL were injected and eluted by phosphate buffered saline (PBS) containing EDTA (1 mM) at a flow rate of 0.5 ml/min. Fractions of 50 or 150 µL were collected and mixed with 100 µL cholesterol reagent (CHOD-PAP, Roche Diagnostics, US), left 20 min at 37^0^C and finally read at an absorbance of 500 nm in the spectrophotometer. Fractions collected were very-low density lipoprotein (VLDL), low-density lipoprotein (LDL) and high-density lipoprotein (HDL).

*Tissue handling, en face evaluation, histology and histomorphometry*

For euthanasia, animals were bled under general anesthesia. Immediately following euthanasia, the thorax was opened and the heart excised and processed as described below.

Coronary arteries

Left and right coronary arteries were manually flushed with 60 ml of isotonic saline, followed by 60 ml of 10% neutral-buffered formalin. The left anterior descending (LAD) branch of the left coronary artery was trimmed into max fourteen 2 mm segments from the origin of the artery and distally, as previously described [3]. Each segment was immersion-fixed in 10% neutral-buffered formalin for 24 hours followed by dehydration and paraffin-embedding.

Histology

Three µm paraffin sections from the aortas and LADs were dewaxed and rehydrated using double-distilled H_2_O. The aorta sections were stained with Movat’s Pentachrome and haematoxylin and eosin (H&E) for evaluation of morphology. LAD sections were stained with Verhoeff’s Van Gieson and H&E, respectively, for visualization of the internal and external elastic lamina, and overall morphology. Lesion morphology in the aorta and the LAD was classified according to modified human guidelines and previous findings in pigs (see Table 1) [4-6]. Coronary plaque burden was quantified as coronary plaque area (CPA) in µm^2^ and intima/media-ratio (Ratio), assessed using an image analysis program (VIS^TM^, DK). Reported values were averaged values of CPA and Ratio of all intact LAD segments (up to six per animal). All observations were performed blinded.

Aorta

The entire aorta was excised, opened ventrally at the long axis and immersion-fixed flat in 10% neutral-buffered formalin, for a minimum of 24 hours. To evaluate plaque area *en face* in the entire aorta, lipophilic staining (Sudan IV, Alfa Aesar, Karlsruhe, GE) was applied and aortic plaque area (APA) evaluated using image analysis software (VIS^TM^, Visiopharm, Hoersholm, DK), as described previously [4]. A segment 3 mm below the exit of the left renal artery was excised, dehydrated and paraffin-embedded for histology.

**Table 1** Histopathological scoring system of intimal lesions in minipig model of atherosclerosis, with increasing lesion severity from top to bottom of the table. The scoring is modified after Virmani *et al*, and previous findings in pigs [4-6].

| **Classification** | **Description** | **Details** |
| --- | --- | --- |
| **Non-atherosclerotic intimal lesions** | Intimal thickening | No overt foam cells, but intimal thickening |
|  | Simple xanthoma | Abundant foam cells, or lipid-loaded smooth muscle cells. |
|  | Xanthoma with deposits of fibrous tissue and/or calcification | Fibrous tissue and/or calcification in the intima, observed in addition to the above mentioned findings |
| **Progressive atherosclerotic lesions** | Pathological intimal thickening | Presence of extracellular intimal lipid (cholesterol clefts), with or without calcification and/or fibrous tissue deposits |
|  | Fibroatheroma | Necrotic areas and/or necrotic core with thick fibrous cap delineating necrotic areas/core from lumen |
|  | Thin-capped fibrous atheroma | Necrotic core with thin fibrous cap delineating necrotic core from lumen |

**References**

1. Larsen MO, Wilken M, Gotfredsen CF, Carr RD, Svendsen O, Rolin B: Mild streptozotocin diabetes in the Göttingen minipig. A novel model of moderate insulin deficiency and diabetes. Am J Physiol Endocrinol Metab 2002;282(6):E1342-E1351.

2. Christoffersen BO, Gade LP, Golozoubova V, Svendsen O, Raun K: Influence of castration-induced testosterone and estradiol deficiency on obesity and glucose metabolism in male Gottingen minipigs. Steroids 2010;75(10):676-84.

3. Kim DN, Schmee J, Lee KT, Thomas WA: Atherosclerotic lesions in the coronary arteries of hyperlipidernic swine Part 1. Cell increases, divisions, losses and cells of origin in first 90 days on diet. Atherosclerosis 1987;64(2–3):231-242.

4. Al-Mashhadi RH, Sorensen CB, Kragh PM, Christoffersen C, Mortensen MB, Tolbod LP, Thim T, Du Y, Li J, Liu Y, Moldt B, Schmidt M, Vajta G, Larsen T, Purup S, Bolund L, Nielsen LB, Callesen H, Falk E, Mikkelsen JG, Bentzon JF: Familial Hypercholesterolemia and Atherosclerosis in Cloned Minipigs Created by DNA Transposition of a Human PCSK9 Gain-of-Function Mutant. Sci Transl Med 2013;5(166):166ra1.

5. Virmani R, Kolodgie FD, Burke AP, Farb A, Schwartz SM: Lessons from sudden coronary death: a comprehensive morphological classification scheme for atherosclerotic lesions. Arterioscler Thromb Vasc Biol 2000;20:1262-1275.

6. Thim T, Hagensen MK, Drouet L, Bal dit S, Bonneau M, Granada JF, Nielsen LB, Paaske WP, Botker HE, Falk E: Familial hypercholesterolaemic downsized pig with human-like coronary atherosclerosis: a model for preclinical studies. EuroIntervention 2010;6:261-268.
