# Supplementary material for: Göttingen minipig model of diet-induced atherosclerosis: influence of mild streptozotocin-induced diabetes on lesion severity and markers of inflammation evaluated in obese, obese and diabetic, and lean control animals
Source: J Transl Med. 2015 Sep 22;13:312. doi: 10.1186/s12967-015-0670-2 (PMC4580291; doi:10.1186/s12967-015-0670-2)
Supplement: Supplementary file 2 — Additional file 2. Supplementary results. [file 12967_2015_670_MOESM2_ESM.docx]

**Additional file 2: Supplementary results**

**Table 1** Statistical findings of circulating inflammatory markers effect on aortic and coronary plaque area.

| Response variable | Explanatory variable* | Regression coefficient | SE | *p*-value |
| --- | --- | --- | --- | --- |
| CPA† | PAI-1 | 0.000045 | 0.0000021 | 0.0481 |
|  | oxLDL | 0.0127 | 0.0068 | 0.0235 |
|  | Group | - | - | 0.0001 |
| Ratio† | Group | - | - | <0.0001 |
| APA† | oxLDL | 0.0150 | 0.0070 | 0.0395 |
|  | Group | - | - | <0.0001 |

* The response variables coronary plaque area (CPA), intima/media ratio (Ratio) and aortic plaque area (APA) were evaluated in ANOVA with inflammatory markers (oxidized LDL (oxLDL), C-reactive protein (CRP), plasminogen activator inhibitor-1 (PAI-1)) and group as explanatory variables. Using stepwise backwards reduction, significant findings (*p*<0.05) are displayed.
†Log transformed
Abbreviations: SE: Standard error. LDL: Low-density lipoprotein.

**Table 2** Statistical findings of circulating lipid markers effect on aortic and coronary plaque area.

| Response variable | Explanatory variable* | Regression coefficient | SE | *p*-value |
| --- | --- | --- | --- | --- |
| CPA† | Group | - | - | <0.0001 |
| Ratio† | Group | - | - | <0.0001 |
| APA† | TG | 0.2305 | 0.0733 | 0.0039 |
|  | HDL | -0.2562 | 0.1227 | 0.0461 |
|  | Group | - | - | <0.0001 |

*The response variables coronary plaque area (CPA), intima/media ratio (Ratio) and aortic plaque area (APA) were evaluated in ANOVA with lipid markers (triglycerides (TG), very-low density lipoproteins (VLDL), high-density lipoproteins (HDL), low-density lipoproteins (LDL) and group as explanatory variables. Using stepwise backwards reduction with significant findings (p<0.05) displayed.
†Log transformed
Abbreviations. SE: standard error

**Table 3** Study duration effect on aorta lesion severity in high-fat/high-cholesterol fed animals (HFD) at diet-week 22 (n=6) and 43 (n=5). Graduation of lesions according to Virmani *et al*.[1] No statistical significant difference was observed in Fisher’s exact test (significance level p<0.05)

| Diet week |  | Non-atherosclerotic intimal lesions | Progressive atherosclerotic lesions |  | **N Total** |
| --- | --- | --- | --- | --- | --- |
| 22 |  | 3 | 3 |  | 6 |
| 43 |  | 2 | 3 |  | 5 |
| **Total** |  | 5 | 6 |  |  |

Abbreviation: HFD, High-fat/high-cholesterol diet fed animals

**References**

1. Virmani R, Kolodgie FD, Burke AP, Farb A, Schwartz SM: Lessons from sudden coronary death: a comprehensive morphological classification scheme for atherosclerotic lesions. Arterioscler Thromb Vasc Biol 2000; 20:1262-1275.
